# An Unusual Case of Thigh Swelling

**DOI:** 10.7759/cureus.37662

**Published:** 2023-04-16

**Authors:** Sarah Douglas-Seidl, Ramesh Damodaran Prabha, Yasser Farooque

**Affiliations:** 1 General Surgery, Liverpool Hospital, Sydney, AUS; 2 General Surgery, Gold Coast University Hospital, Gold Coast, AUS

**Keywords:** leg swelling, vascular malformations, cystic lymphangioma, lower extremity tumour, thigh mass

## Abstract

Lymphangiomas are benign tumours, almost exclusively found in children. Primary work-up includes imaging. We report a case of lymphangioma in the leg in an adult patient, initially masked as a myxoma. Our patient underwent ultrasound, computerised tomography, and magnetic resonance imaging, which were suggestive of myxoma. Treatment for lymphangioma varies from sclerotherapy to definitive surgical management. In our case, surgical management was selected under consideration of myxoma; however, histopathology confirmed lymphangioma. Lymphangiomas in adult patients can be masked by other conditions and should be considered as a differential in lower leg swellings.

## Introduction

Cystic lymphangiomas are rare, slow-growing, benign tumours of the lymphatic system. They more commonly occur in the head and neck area in 75% of cases, and less commonly in the extremities [1,2]. Most cases of cystic lymphangiomas have been reported in children as congenital malformations [3]. They are usually asymptomatic but can appear on the skin as red or bluish dots and progress into a palpable mass. Imaging like ultrasound (US), computerised tomography (CT), and magnetic resonance imaging (MRI) can be helpful for delineating the extent of involvement. The use of a single imaging diagnostic method has been described previously, where surgery was conducted following primary diagnosis with US [4]. The diagnosis of cystic lymphangioma is confirmed by histopathology. Treatment of cystic lymphangiomas is dependent on the characteristics like size, location, and the patient’s symptoms. The standard of care is surgical excision [5].

## Case presentation

**Figure 1:**
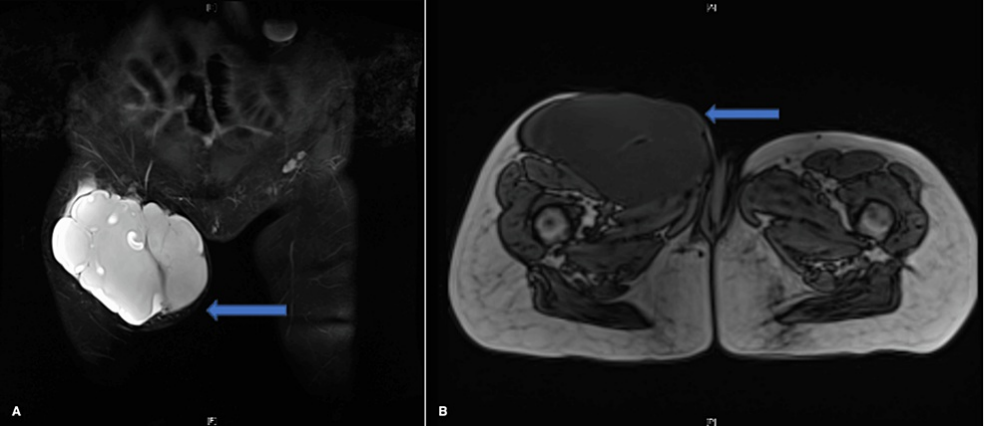
MRI of right thigh lesion A: Coronal view of thigh lesion. Well-defined multi-septated mass measuring 14 x 7.6 x 11.4 cm, deep to the skin, and separate from deep fascia and anterior medial right thigh with compression of the anterior compartment muscles. Heterogenous marrow pattern is demonstrated with enhancement of uncertain nature. B: Axial view of thigh lesion

A 62-year-old female presented with swelling on the thigh without any underlying trauma or surgery. The patient was noted to have a right thigh mass over the past four years prior to presentation with an increase in the size of the mass and discomfort over a period of six months prior to presentation. Her other medical history consisted of cervical cancer, awaiting hysterectomy. On physical examination, the mass was soft, mobile, and measured 12 x 11 cm in size without any palpable inguinal lymph nodes and no sensory or motor deficits was identified. USG showed a right thigh mass measuring 12 x 11 x 6.8 cm. MRI was consistent with a 14 x 11.4 x 7.6 cm well-defined, multi-septated mass. Pre-operative CT scan showed a soft tissue mass in the anterior compartment of the right thigh measuring 15.4 x 12.8 x 9.6 cm. Both CT and MRI were suggestive of anterior thigh myxoma (Figure 1). 

**Figure 2:**
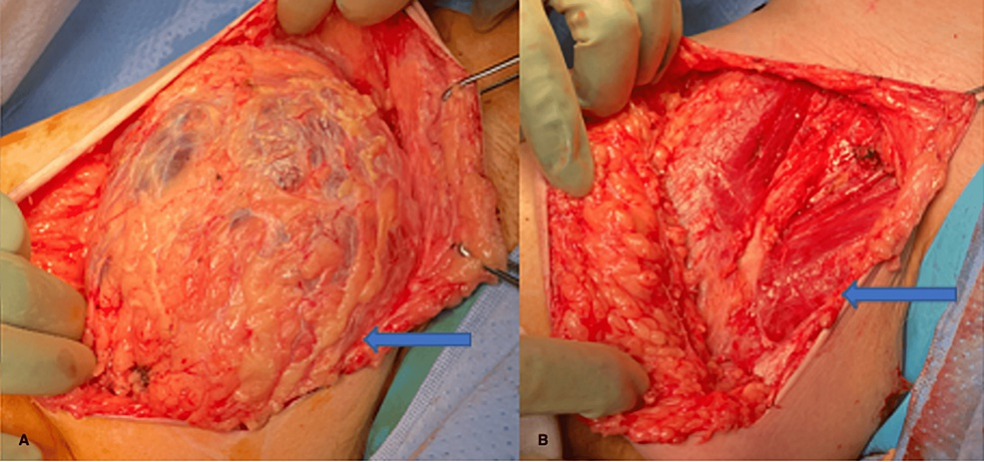
Intra-operative image of lesion before and after surgical-excision A: Blue arrow showing lesion before excision. B: Blue arrow showing structure after resection of lesion.

The patient underwent surgery under general anaesthesia. Superior and inferior skin flaps were raised. An encapsulated subcutaneous mass was found and dissected off the deep thigh fascia. There was no muscular involvement. Involvement of a branch of the superficial femoral artery was noted and suture ligation was performed prior to excision. The superficial femoral vein and femoral triangle with femoral vessels underneath were not involved (Figure 2). 

After the excision, a Jackson-Pratt (JP) drain and Yates drain were inserted, which were sequentially removed in the week after surgery. The histopathology confirmed benign cystic lymphangioma. The patient did not have any postoperative complications. 

**Figure 3:**
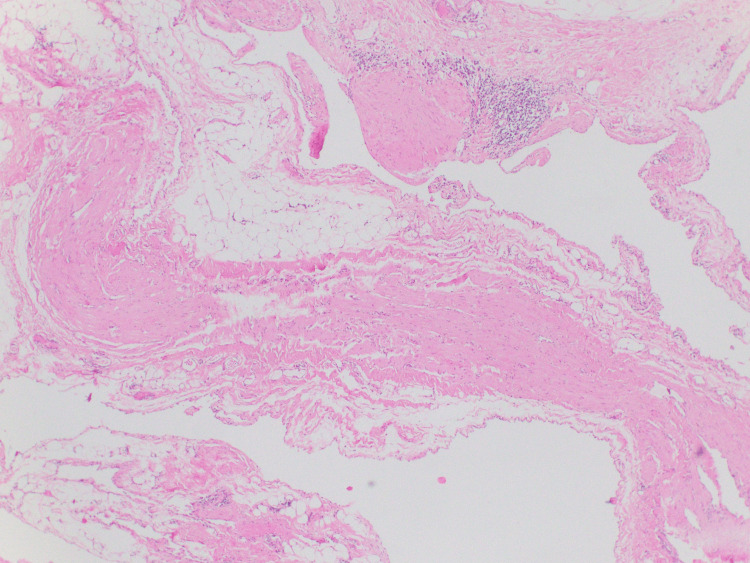
Histopathology confirming diagnosis of lymphangioma (hemotoxylin and eosin staining, x10) Histopathology showing multiple irregular spaces, endothelial cell lining, small vascular channels and lymphocytic accumulation.

## Discussion

The finding of cystic lymphangioma on the lower extremity in an adult patient is extremely rare, especially in the absence of trauma or any other injury [6,7]. Cases have been almost exclusively reported in children [6,7]. 

In the literature, a case of lymphangioma on the thigh in an adult was reported, occurring post-trauma after an accident with progressive worsening over a period of 20 years [8]. A further case with cystic lymphangioma in the lower extremity was reported in the popliteal fossa of a 30-year, masquerading as Baker's cyst, treated by excision [9]. One case has been described of a young adult with increasing leg swelling since childhood consistent with the diagnosis of lymphangioma in adulthood [10].

Surgical excision shows the lowest recurrence rate contrary to other treatment options for cystic lymphangioma [5]. However, surgical excision can be difficult if there is muscular involvement [5]. Newer treatment options include sclerotherapy with bleomycin or Picibanil (OK-432), which can be used as an alternative to excision. OK-432 injection has shown to be an effective treatment for macrocytic lymphatic lesions of 2 cm or more, independent of the location and ineffective in smaller lesions [11]. Percutaneous image-guided sclerotherapy with doxycycline has been effectively used for non-resectable lymphangiomas [12]. In children, aspiration of fluid in unilocular cystic lymphangiomas has been previously reported as helpful prior to definitive surgical excision [6]. 

## Conclusions

Lymphangiomas of lower limbs are extremely rare presentations in adults and should be included in the differential diagnosis in the work-up of mass lesions of lower extremities. They can be easily masked by other pathologies, which can lead to inappropriate management decisions. However, in our case, the management was suitable for lymphangioma despite a masked diagnosis.
